# Contrast-enhanced CMR in patients after percutaneous closure of the left atrial appendage: A pilot study

**DOI:** 10.1186/1532-429X-13-33

**Published:** 2011-07-04

**Authors:** Oliver K Mohrs, Nina Wunderlich, Steffen E Petersen, Anselm Pottmeyer, Hans-Ulrich Kauczor

**Affiliations:** 1 Darmstadt Radiology, Dpt. of Cardiovascular Imaging at Alice-Hospital, Dieburger Strasse 29-31, D-64287 Darmstadt, Germany; 2 University of Heidelberg, Dpt. of Diagnostic and Interventional Radiology, Im Neuenheimer Feld 110, D-69120 Heidelberg, Germany; 3 Cardio-Vascular Center (CVC), Sankt Katharinen, Seckbacher Landstrasse 65, D-60389 Frankfurt, Germany; 4 Centre for Advanced Cardiovascular Imaging, William Harvey Research Institute, Barts and The London NIHR Biomedical Research Unit, The London Chest Hospital, Bonner Road, London, E2 9JX, UK

## Abstract

**Background:**

To evaluate the feasibility and value of first-pass contrast-enhanced dynamic and post-contrast 3D CMR in patients after transcatheter occlusion of left atrial appendage (LAA) to identify incorrect placement and persistent leaks.

**Methods:**

7 patients with different occluder systems (n = 4 PLAATO; n = 2 Watchman; n = 1 ACP) underwent 2 contrast-enhanced (Gd-DOTA) CMR sequences (2D TrueFISP first-pass perfusion and 3D-TurboFLASH) to assess localization, artifact size and potential leaks of the devices. Perfusion CMR was analyzed visually and semi-quantitatively to identify potential leaks.

**Results:**

All occluders were positioned within the LAA. The ACP occluder presented the most extensive artifact size. Visual assessment revealed a residual perfusion of the LAA apex in 4 cases using first-pass perfusion and 3D-TurboFLASH indicating a suboptimal LAA occlusion.

By assessing signal-to-time-curves the cases with a visually detected leak showed a 9-fold higher signal-peak in the LAA apex (567 ± 120% increase from baseline signal) than those without a leak (61 ± 22%; p < 0.03). In contrast, the signal increase in LAA proximal to the occluder showed no difference (leak 481 ± 201% vs. no leak 478 ± 125%; p = 0.48).

**Conclusion:**

This CMR pilot study provides valuable non-invasive information in patients after transcatheter occlusion of the LAA to identify correct placement and potential leaks. We recommend incorporating CMR in future clinical studies to evaluate new device types.

## Background

Atrial fibrillation is the most common sustained cardiac arrhythmia and affects 5 percent of people older than 65 years and 10 percent older than 75 years [1]. It represents a major risk factor for ischemic cerebral stroke or peripheral embolism, especially due to embolism of thrombi forming in the left atrial appendage (LAA). LAA is the main location for left atrial thrombus formation related to the phenomenon of atrial stunning [2]. Anticoagulation is required to prevent further cerebral events as patients in atrial fibrillation have a 5-fold higher risk of embolic stroke than those in sinus rhythm [3-5].

However, long-term anticoagulation is frequently associated with problems of safety and tolerability, such as increased risk of bleeding. Occlusion of the left atrial appendage could be a potential alternative strategy for prophylaxis of embolism. In comparison to surgical amputation, the percutaneous transcatheter occlusion of LAA is a minimally invasive technique and yields promising results in animal [6] and human studies [7-13].

Currently, following the transcatheter procedure, a chest X-ray is performed to confirm the correct placement of the occluder and transesophageal echocardiography allows for the evaluation of possible thrombotic appositions on the device. To date, it has been still challenging to assess directly and non-invasively the residual LAA perfusion indicating a persistent leak due to an insufficient design, a mismatch of device and LAA anatomy or a failure of complete coverage of the atrial-facing surface of the device with neoendothelial-like cells [7]. A persistent leak detected by CMR may be important as this could indicate a remaining risk of thromboembolism from the LAA.

The purpose of this pilot study was to evaluate the feasibility and value of first-pass contrast-enhanced dynamic and post-contrast 3D CMR in patients after transcatheter occlusion of LAA to identify incorrect placement and persistent leaks.

## Materials and methods

### Study Population

**Figure 1:**
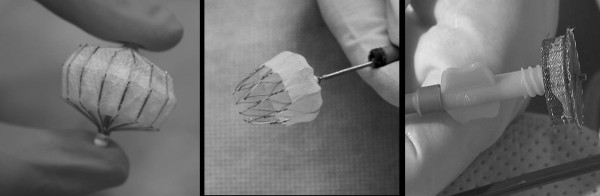
**Different LAA occluder device types**. Common criteria are transcatheter transseptal delivery via femoral venous access, self-expanding nitinol-made cage or mesh, designed to be permanently implanted at or slightly distal to the LAA ostium to trap potential emboli before they exit the LAA. **A (left panel) **shows the historically first LAA device named PLAATO (Appriva Medical Inc.) with a nitinol-made cage covered with an polytetrafluoroethylene membrane with small anchors along the struts; range of diameters 15-32 mm. Faced to the apex of left atrial appendage the cover is opened for the filling of blood and a later thrombosis inside the device. **B (middle panel) **shows the Watchmen device (Atritech Inc.) with a polyethylene membrane on the atrial-faced surface of a nitinol-made cage and a row of fixation barbs; range of diameters 21-33 mm. **C (right panel) **ACP device (AGA Medical Inc.) is constructed from a nitinol mesh and polyester patch, consists of a lobe and a disc connected by a central waist; range of diameters 16-30 mm.

7 adult patients (mean age 68 ± 8, range 56-78 years; 2 females and 5 males) with non-rheumatic atrial fibrillation received transcatheter occlusion of LAA due to warfarin contraindications (intracerebral bleeding, thomboembolic events despite warfarin therapy, exanthema). 4 patients received a PLAATO device (eV3 Inc., Minneapolis, MN, USA), 2 patients a Watchman device (Atritech Inc.; Minneapolis; MN, USA) and one patient an ACP device (AGA Medical Inc., Plymouth, MN, USA). Our study population for this pilot study was drawn from subsequent patients undergoing transcatheter occlusion of LAA with clinically suspected device malposition or residual leaks who consented to partake in this CMR study. The different designs of the devices are displayed in Figure 1.

The patients underwent CMR median 102 days (range 91-229 days) after the LAA occlusion procedure after written informed consent had been obtained. None of the patients had suffered a stroke between the transcatheter occlusion of LAA and the date of the CMR scans. The study was approved by the local ethics committee.

### Magnetic Resonance Imaging

Magnetic resonance imaging was performed on a 1.5-T CMR system (MAGNETOM Avanto with SQ-engine gradients, Siemens AG, Healthcare Sector, Germany). For signal detection, the combinations of a six-channel body matrix coil and six elements of a spine matrix coil were used. The ECG-signal was received via an external system (Magnitude 3150, InVivo Research Inc., Orlando, FL, USA).

ECG-gated segmented steady-state-free-precession (SSFP) cine-sequences (TR = 2.7 ms, TE = 1.2 ms, temporal resolution 34 ms, voxel-size = 1.7 × 1.3 × 6.0 mm^3^) served to determine LAA anatomy and for detection of the occluder system.

Two slices of a saturation-recovery SSFP sequence (TrueFISP, TE = 2.7 ms, TI = 217 ms, flip angle 50°, temporal resolution 832 ms, matrix 144 × 256, voxel-size = 1.8 × 1.4 × 6.5 mm^3^) were performed simultaneously planned from the optimal views on cine studies in the oblique axial and sagittal long axis. For each slice 40 consecutive images were acquired during the administration of a bolus of Gadoterate Meglumin (0.1 mmol/kg bodyweight Gd-DOTA, Dotarem, Guerbert) followed by 30 ml saline, both at 4 ml/second into an antecubital vein.

After the administration of another 0.1 mmol/kg bodyweight Gd-DOTA (Dotarem, Guerbet) an ECG-gated 3D-TurboFLASH sequence (TE = 1.5 ms, TI was optimized [typical values 200-350 ms], minimum acquisition window typically 451 ms, flip angle 10°, matrix 152 × 256, number of slices = 12, voxel size = 1.9 × 1.4 × 4.0 mm^3^) was acquired in the same optimized planes.

### Image analysis

The data sets (cine localizer sequences, dynamic first pass perfusion and post-contrast 3D images) were evaluated by consensus of 2 observers experienced in cardiovascular radiology. The readings were performed blinded to any clinical information. In every patient the readers evaluated qualitatively for occluder size (including surrounding metal-related, artificial signal void), occluder localization (judged as either outside LAA, at the LAA ostium or deep into the LAA body) and persistent leaks (contrast enhancement of the LAA apex distal to the occluder device).

Additionally, contrast-enhanced perfusion studies were analyzed generating signal-time-curves (using Syngo "mean curve"; Siemens AG healthcare, Germany). Two different regions of interest (ROIs) were placed in the left atrium and in the LAA apex distal to the occluder device. Due to heart movement ROIs were manually fitted to every image without changing the size. The signal-time-curves were normalized to baseline signal (second image, as the signal in the first image was not fully saturated after the saturation-preparation pulse) for each ROI [14]. The single data points represent the percentage signal increase compared to the baseline signal.

### Statistical Analysis

Statistical analysis was performed using SPSS 18.0. Quantitative data are presented as mean and standard deviation or median and range when appropriate. The independent samples Mann Whitney U Test (non-parametric test) was performed to assess for differences in time to the signal peaks in the left atrium and the LAA apex and signal peaks as a percentage to baseline signal. A p value less than 0.05 was considered statistically significant.

## Results

### Sizes and localization of occluder devices

There was no dislocation of the devices outside the LAA. In 4 cases the atrial-facing device border was localized at the ostium of LAA whereas in 3 cases the device was localized more deeply within the LAA body. All of the 3 cases with device localization more deeply within the LAA presented a leak but only one localized at the ostium of LAA. In 5 cases the occluder-related signal voids show a slight bulging (4.2 ± 3.6 mm) into the left atrium. The mean size of the occluder device including surrounding artefacts was 28.6 × 25.3 × 25.4 ± 3.9 × 3.3 × 3.1 mm.

**Figure 2:**
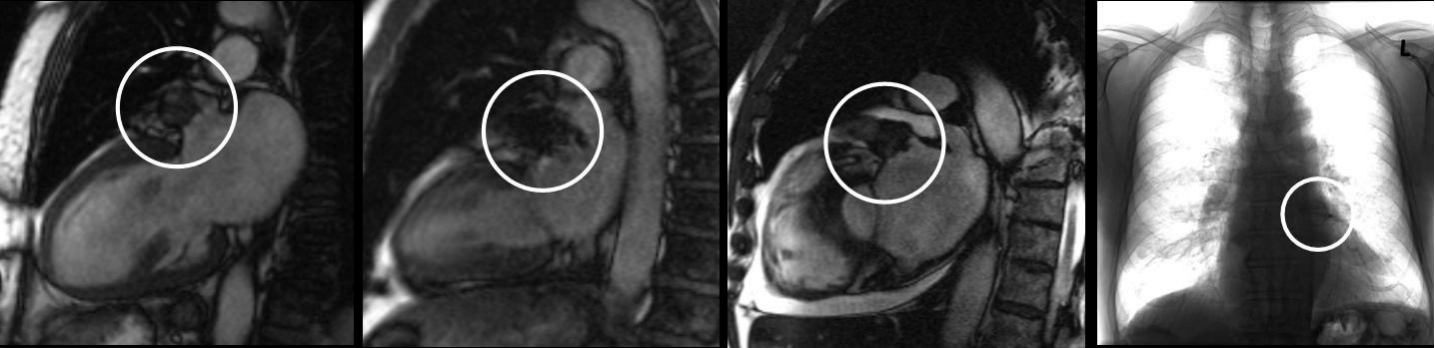
**Different appearance of LAA devices**. Cine TrueFISP-SSFP sequences demonstrate signal voids within LAA representing the correct placement and the exclusion of a migration of the devices: **1A (left panel) **PLAATO device; **1B **Watchmen device and **1C **ACP device. For comparison figure 1D **(right panel) **shows a chest-X-ray of a PLAATO device.

Baseline characteristics of each patient are presented in table 1. Figure 2 illustrates the different types and CMR-related aspects of devices.

**Table 1:** Baseline characteristics

**No**.	Device type	Real device diameter [mm]	CMR device signal void [mm]	CMR device localization	Residual leak	Time to left atrial signal peak [heartbeats]	Left atrial signal peak [% from baseline]	Time to left atrial appendage signal peak [heartbeats]	Left atrial appendage signal peak [% from baseline]
1	PLAATO	26	30 × 24 × 24	LAA body	Yes	22	582	29	519

2	PLAATO	29	31 × 27 × 27	LAA body	Yes	18	550	24	502

3	PLAATO	32	31 × 29 × 29	LAA ostium	No	32	249	10	39

4	PLAATO	29	31 × 27 × 27	LAA body	Yes	16	302	25	746

5	ACP	22	31 × 28 × 28	LAA ostium	No	20	602	25	83

6	Watchman	22	22 × 21 × 21	LAA ostium	No	26	592	29	61

7	Watchman	27	24 × 21 × 22	LAA ostium	Yes	23	476	35	499

### Assessment of residual leaks

Visual evaluation of contrast-enhanced first-pass perfusion CMR revealed contrast enhancement at the apex of the LAA beyond the device indicating a residual leak in 4 cases.

Patients with visually detected residual perfusion showed 9-fold higher peaks in the LAA apex distal to the device (567 ± 120% from baseline signal) than those without a leak (61 ± 22% from baseline signal; p < 0.03). In contrast, the signal peaks in the left atrium body showed no difference in patients with compared to patients without leaks (leak 481 ± 201% vs. no leak 478 ± 125%; p = 0.48).

The time to signal peaks was similar in patients with and without leaks in the left atrium (leak 26 ± 6 heartbeats vs. no leak 20 ± 3 heartbeats; p = 0.16) or in the LAA (leak 21 ± 10 heartbeats vs. no leak 28 ± 5 heartbeats; p = 0.47).

According to the anatomical size the median pixel size of ROIs in the left atrium (58 pixel; range 37-221 pixel) was larger than in the left atrial appendage (21 pixel; range 12-34 pixel).

**Figure 3:**
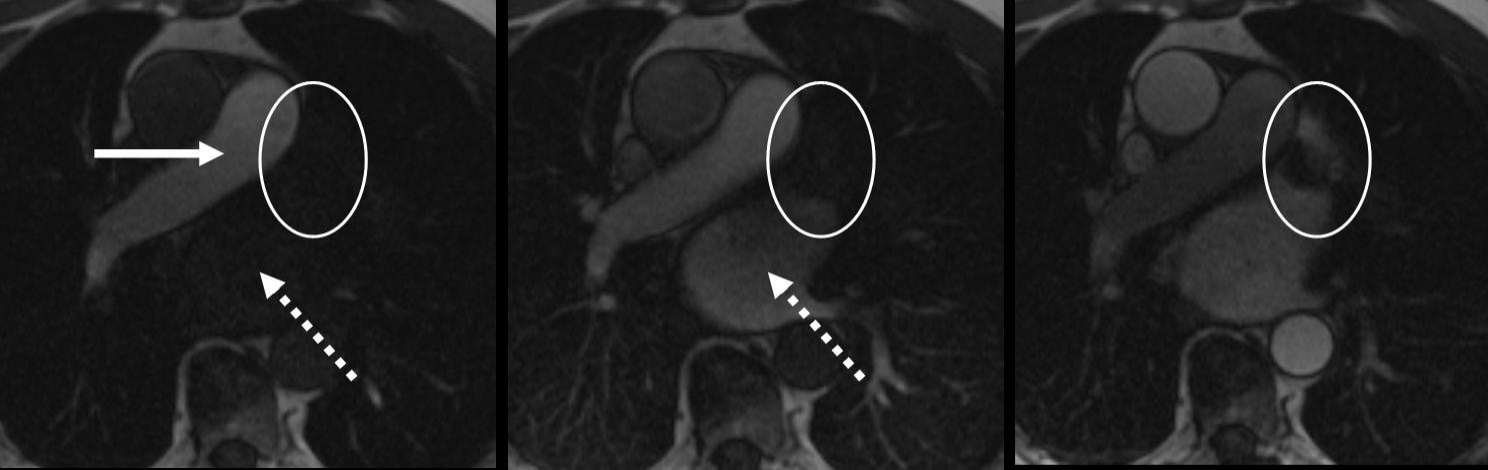
**67-year-old male after percutaneous transcatheter LAA occlusion using a Watchman device**. Figures 3A-D show the temporal sequence of first-pass contrast-enhanced perfusion imaging (see also movie 1 and 2). **A (left): **contrast-enhancement of the pulmonary trunk (arrow) but not in the left atrium (dotted arrow); typical signal loss at the framework of the device (circle). **B (middle): **contrast-enhancement in left atrium (dotted arrow) but not inside the device or at the apex of left atrial appendage (circle). **C (right): **contrast-enhancement of the LAA apex beyond the device representing a slow wash-in due to a small residual leak.

**Figure 4:**
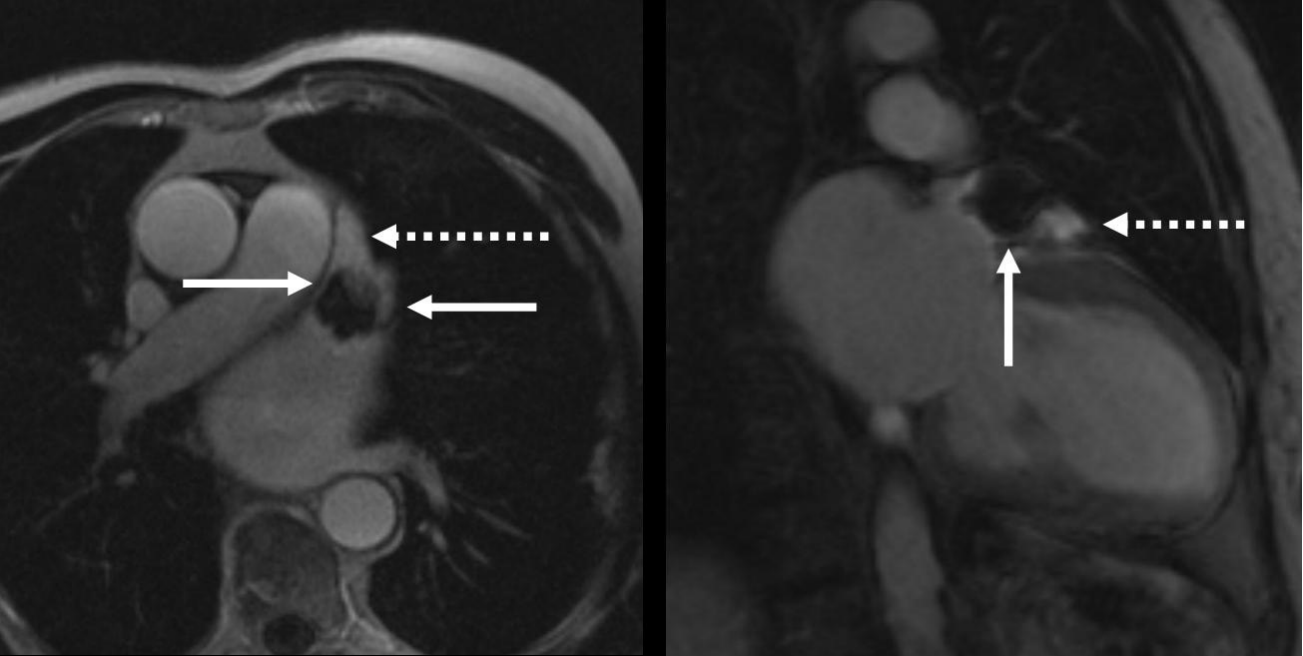
**Comparison of a post-Gadolinium 3D TurboFLASH sequence in patients with an insufficient coverage of the LAA after transcatheter LAA occlusion using a Watchmen device (4A left) and a PLAATO device (4B right)**. Note the enhancement around the margins (arrows) and of the LAA apex beyond the device (dotted arrows) indicating a residual leak.

**Figure 5:**
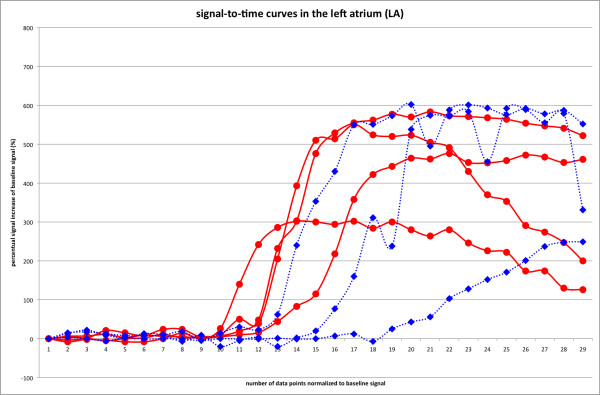
**Comparison of normalized signal-time curves of all 7 patients in the left atrium**. As expected, LA signal-time curves show a similar pattern of enhancement in patients without (blue dashed lines with diamond symbols) and with (red solid lines with dot symbols) sufficient coverage (i.e. with leak) of the transcatheter occlusion of the LAA.

**Figure 6:**
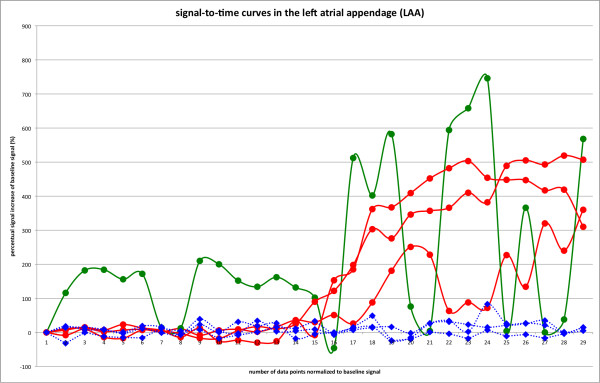
**Comparison of normalized signal-time curves of all 7 patients in the left atrial appendage**. LAA signal-time curves behave in two patterns: No discernible peaks can be seen in normalized signal-time curves in patients with sufficient LAA occlusion and peaks of at least 200% compared to normalized baseline can be seen in those without sufficient coverage of the transcatheter occlusion of the LAA. All signal-time curves start with the second heartbeat which was used for normalization of the signal-time curves (the first image was not used due to incomplete saturation). Variations in the erratic signal-time curve (green solid line with dot symbols) likely represents partial volume effects due to suboptimal breath holding or arrhythmia (atrial fibrillation). The solid curve in the right panel with two positive peaks in the early part of the signal time curve are likely due to such a partial volume effect, as the maximal possible region of interest which could be drawn consisted of only 12 pixels. The differences in magnitude of the peak signal could be caused by individual contrast geometry variations."

Cases of visually detected residual leaks after transcatheter LAA occlusion are presented in Figure 3 and 4. Please also see movies 1 and 2 (additional file 1 and 2) which show the temporal sequence of first-pass contrast-enhanced perfusion imaging. Figure 5 and 6 compare signal-to-time curves in patients without and with a residual perfusion of the LA (Figure 5) and LAA (Figure 6).

## Discussion

Our pilot study highlights the potential role of contrast enhanced CMR to evaluate the localization of LAA occluder systems and to detect residual leaks after implantation.

Atrial fibrillation affects 3-5% in patients older than 65 years and is responsible for 15-20% of strokes [15]. Due to the risk of thromboembolism many patients with atrial fibrillation are treated with anticoagulants. But long-term therapy with warfarin is associated with an increased risk of minor (5 to 10% per year) and major (1-2% per year) hemorrhagic complications [16]. This underlies the rationale to establish alternative approaches, such as transcatheter percutaneous LAA occlusion. Research study protocols define successful treatment as correct placement of the device at or slightly distal to the LAA ostium, appropriate implant size by measuring the deployed diameter of the implant in situ and the absence of residual LAA perfusion.

Currently, chest X-ray and echocardiography are used after transcatheter procedure to verify correct placement of the device and to exclude thrombotic appositions or residual leaks. But chest X-ray is only able to detect extracardiac displacement and echocardiography is limited to evaluate residual flow since flow velocities within LAA in patients with atrial fibrillation are highly variable even before the procedure [17]. Pre-procedural measurements of LAA by transesophageal echocardiography do not always accurately predict the size of LAA occlusion devices and it is still unclear how accurately transesophageal echocardiography is able to confirm the correct placement of the device during and after the procedure [18]. Also, the major drawback of transesophageal echocardiography is its semi-invasiveness and the need for sedation in many cases.

There are age- and sex-related differences in LAA dimensions as well as various anatomical variants [19,20]. Accurate measurement of LAA diameters is very challenging and probably high-resolution contrast-enhanced cardiac CT will play a major role prior to transcatheter procedures in the future [18]. However, CMR offers non-invasively and without any radiation a reliable evaluation of post-procedural localization and potential residual leaks of the device as documented in our study. Currently, for safety concerns a time interval of 6 weeks between implantation and CMR examination is recommended for intracardiac devices to ensure that the device is fixed to the endocardial tissue [21]. In general, LAA occluder devices are safe, especially nitinol-based intracardiac devices [22].

Over- or undersizing, device migration or displacement could contribute to unsuccessful device deployment. Incomplete occlusion of LAA is one of the major concerns leaving the clinician in the dilemma of whether it is safe to stop warfarin therapy. CMR-based measurements could be helpful to understand the principle of residual leaks due to potential for suboptimal device deployment. All devices with residual perfusion were localized deeper into the LAA and a small rim of contrast agent could be identified around the margins of the device using 3D imaging sequences.

Diagnosis of post-procedural leaks seems to be detectable using qualitative visual assessment, which obviates the need for time-consuming post-processing. However, less experienced observers may wish to confirm the visual assessment by semi-quantitative assessment and we identified 9-fold higher signal intensity peaks. A possible underlying problem of using our proposed CMR perfusion technique needs to be considered in the context of devices causing metal artifacts which can lead to spurious local increase of signal in the LAA region before contrast arrival and/or possible loss of contrast related signal after its arrival in the test region.

## Limitations

The high incidence of residual leaks after transcatheter LAA occlusion in our pilot study result is due to a selection bias as we only included patients in whom problems of device malposition or residual leaks were suspected and does not contradict the very promising results of this therapy approach [7-13]. However, this study was designed to demonstrate the feasibility of CMR to detect a potential occluder dislocation and residual LAA perfusion.

Residual perfusion could be a source of thromboemboli and these patients might be considered for further anticoagulation. However, despite our encouraging initial experience using contrast-enhanced CMR, this warrants further investigation regarding its predictive value and impact on treatment strategies. Given the small numbers of LAA device occlusion performed even in specialist centres, this should be done in a multi-center study.

Our pilot study did not address the important question whether CMR is able to detect small thrombi on the device surface. In this pilot study we did not compare our findings to transoesophageal echocardiography.

Determination of signal time curves without partial volume effect can be difficult or occasionally impossible to obtain due to the complex and variable left atrial appendage anatomy. However, leaks around the left atrial appendage occluder device are usually visible and the signal time curves are most often for confirmation only.

## Conclusions

CMR is feasible to confirm occluder placement and to detect residual LAA leaks non-invasively and without radiation. Given the variable and complex anatomy of the LAA CMR may inform device companies how to tailor and optimize the design of such devices and to test them in small pilot studies. We propose that clinical trials to test LAA occlude devices should incorporate contrast-enhanced CMR.

## Abbreviations

LAA: left atrial appendage; CMR: cardiac magnetic resonance imaging; Gd-DOTA: gadolinium gadoterate meglumin; 3D: three-dimensional; FLASH: fast-low-angle-shot; FISP: Fast Imaging with Steady State Precession; SSFP: steady-state-with-free-precession; ROI: region of interest; CT: computed tomography.

## Competing interests

The authors declare that they have no competing interests.

## Authors' contributions

**OKM - **conception and design; acquisition of data; analysis and interpretation of data; drafting the manuscript; revising the manuscript critically; given final approval. **NW - **conception and design; analysis and interpretation of data; revising the manuscript critically; given final approval. **SEP - **conception and design; analysis and interpretation of data; revising the manuscript critically; given final approval. **AP - **conception and design; analysis and interpretation of data; revising the manuscript critically; given final approval. **HUK - **conception and design; analysis and interpretation of data; revising the manuscript critically; given final approval.

## Supplementary Material

Additional file 1 **Movie of a residual peak in sagital angulation**. 67-year-old male after percutaneous transcatheter LAA occlusion using a Watchman device. The movie shows the temporal sequence of first-pass contrast-enhanced perfusion imaging in sagital angulation. The contrast-enhancement of the LAA apex beyond the device is representing a slow wash-in due to a small residual leak. Click here for file

Additional file 2 **Movie of a residual peak in axial angulation**. Same patient and findings as in additional file 1 (movie 1) but in an axial angulation. Click here for file
